# Congenital adrenal hyperplasia presenting as pelvic inflammatory disease in a phenotypic male: A case report

**DOI:** 10.1097/MD.0000000000018387

**Published:** 2020-01-10

**Authors:** Eunsoo Lim, Ja Young Jeon

**Affiliations:** a Department of Emergency Medicine, Ajou University School of Medicine, Suwon; b Department of Endocrinology and Metabolism, Ajou University School of Medicine, Suwon, Republic of Korea.

**Keywords:** congenital adrenal hyperplasia, myelolipoma, pelvic inflammatory disease

## Abstract

**Rationale:**

Congenital adrenal hyperplasia (CAH) is caused by various enzyme deficiencies, among which 21-hydroxylase (21-OH) deficiency accounts for more than 90% of cases. Neonatal screening became mandatory only a few decades ago. Many patients who were born before this went undiagnosed and some of the severely virilized females were raised as men.

**Patient concerns:**

A 58-year old man with a history of excisional surgery in the external genitalia when he was a toddler presented with three days of dysuria and low abdominal pain.

**Diagnosis:**

The patient's laboratory results showed leukocytosis and elevated C-reactive protein (CRP); thus, the physicians decided to perform a computed tomography (CT) scan. The CT demonstrated pelvic inflammatory disease (PID), left adrenal gland myelolipoma, and a mesenteric mass. Meanwhile, we suspected CAH based on the clinical history and assessed the patient's hormone levels. Seventeen-hydroxyprogesterone (17-OH-PG) was markedly elevated and the patient was diagnosed with classic simple virilizing CAH.

**Interventions:**

Intravenous antibiotics were administered, and positron emission tomography-CT (PET-CT) was performed to evaluate any metastases.

**Outcomes:**

After 2 weeks of antibiotic treatment, CRP decreased to 0.12 mg/dL and PID was resolved. The patient opted for resection of the female genitalia along with the mesenteric and adrenal gland tumors in the near future, and was safely discharged.

**Lessons:**

The adrenal gland myelolipoma was thought to have developed as a result of a longstanding exposure to adrenocorticotropic hormone. There are controversies regarding the management of female genitalia in CAH patients who identify themselves as men. In this case, the physician and patient decided to remove the female genitalia because the surgery for the mesenteric mass was inevitable and there was a possibility of recurrent PID. To our knowledge, this is the first article to report primary mesenteric tumor in a CAH patient to date. In conclusion, patients who were born before neonatal screening for CAH became the mainstay, who are suspected to have CAH from their history, and present with abdominal pain must be diagnosed by performing an imaging study, testing levels of serum 17-OH-PG, and screening for female genitalia and adrenal gland myelolipoma.

## 1 Introduction

Congenital adrenal hyperplasia (CAH) is an autosomal recessive disorder with a worldwide incidence ranging from 1:14,000 to 1:18,000 births.^[1]^ Deficiency of various enzymes results in CAH, including 21-hydroxylase (21-OH), 11-beta hydroxylase, 17-alpha hydroxylase, 3-beta hydroxysteroid dehydrogenase and P450 oxidoreductase; however, 21-OH deficiency accounts for more than 90% of CAH cases.^[2,3]^ Clinical manifestations are also highly diverse, depending on the levels of glucocorticoid, mineralocorticoid, and sex steroids.^[2]^

Neonatal screening for CAH was first done in Alaska in the late 1970s, and it became mandatory in Korea in 2006. ^[2,4]^ Before neonatal screening became the mainstay, some non-fatal, simple virilizing CAH cases were undiagnosed and raised as men due to their cultural environment.^[5]^

Here, we report a case of CAH in a phenotypic male who presented with pelvic inflammatory disease (PID) and was first diagnosed with CAH at 58 years of age.

### 1.1 Case report

A 58-year-old man presented to the emergency department (ED) complaining of 3 days of low abdominal pain and dysuria. The abdominal pain was dull and especially aggravated just before defecating. The patient had previously heard from his parents that the he was born with ambiguous genitalia and some parts of the patient's external genitalia were excised when the patient was three years old. Since then, the patient was raised as male. The patient was married to a woman but had no children.

The height of the patient was 140 cm, and he weighed 50.1 kg and was bald. The patient had tenderness on the lower abdomen. The patient had micropenis and no palpable testis. The patient's urethral orifice was located on the perineum, not at the tip of the glans penis. The blood pressure was 114/72 mmHg and temperature was 37°C. White blood cell count was 15,100/μL, C-reactive protein (CRP) was 12.77 mg/dL, and sodium was 138 mMol/L. Urine analysis showed that the patient had pyuria. The ED physicians decided to perform an abdominal computed tomography (CT) for further evaluation. The CT showed

**Figure 1:**
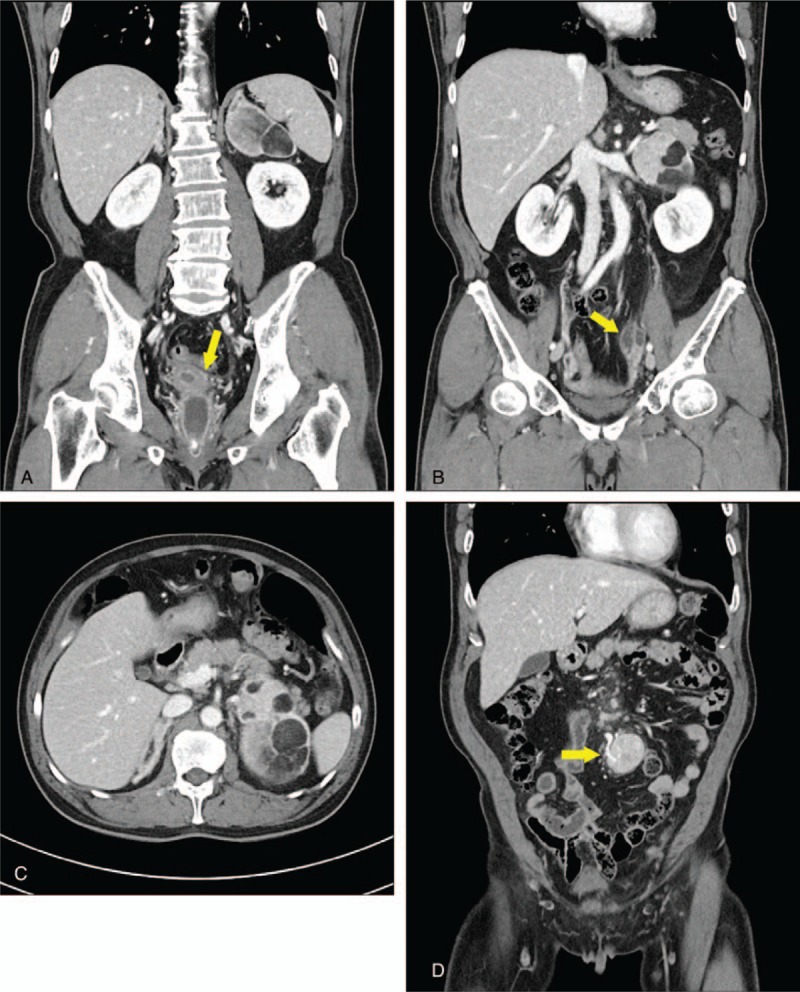
Abdominal computed tomography showing (A) Mullerian structures with fluid collection in the vaginal cavity (arrow); (B) Dilated, thickened left fallopian tube compatible with salpingitis (arrow); (C) A 10 cm myelolipoma in the left adrenal gland; (D) A 3.2 cm hypervascular mass in the small bowel mesentery (arrow).

(1) suspicious PID including left salpingitis;
(2) a 10 cm myelolipoma in the left adrenal gland;
(3) a 3.2 cm hypervascular mass in the small bowel mesentery (Fig. 1).

The patient was admitted to the ED for evaluation of possible CAH and intravenous antibiotic administration of third generation cephalosporin and metronidazole was initiated. After 3 days of antibiotic treatment, CRP decreased to 1.65 mg/dL and the patient reported an improvement of the symptoms although the pain before defecation persisted. Along with antibiotics, a hormonal study regarding CAH was carried out (Table 1). A high 17-OH-progesterone (17-OH-PG) level above 3500 ng/dL suggested that the patient had underlying CAH. Since the patient had ambiguous genitalia from birth and did not have electrolyte imbalance caused by salt wasting, we diagnosed the patient with classic simple virilizing CAH. Virilization of females can be caused by a deficiency in enzymes 21-OH and 11-beta hydroxylase. A deficiency in 11-beta hydroxylase deficiency presents with hypertension and low renin and aldosterone levels; however, the patient was suspected of having 21-OH deficiency due to the normal blood pressure, and normal levels of renin and aldosterone. Although the patient did not show symptoms of adrenal insufficiency, the rapid ACTH test results were abnormal, indicating a cortisol deficiency. During the ACTH stimulation test, response of 17-OH-PG to ACTH was exacerbated. This result further supported the diagnosis of CAH due to a 21-OH deficiency. The results are summarized in Table 2.

**Table 1:**
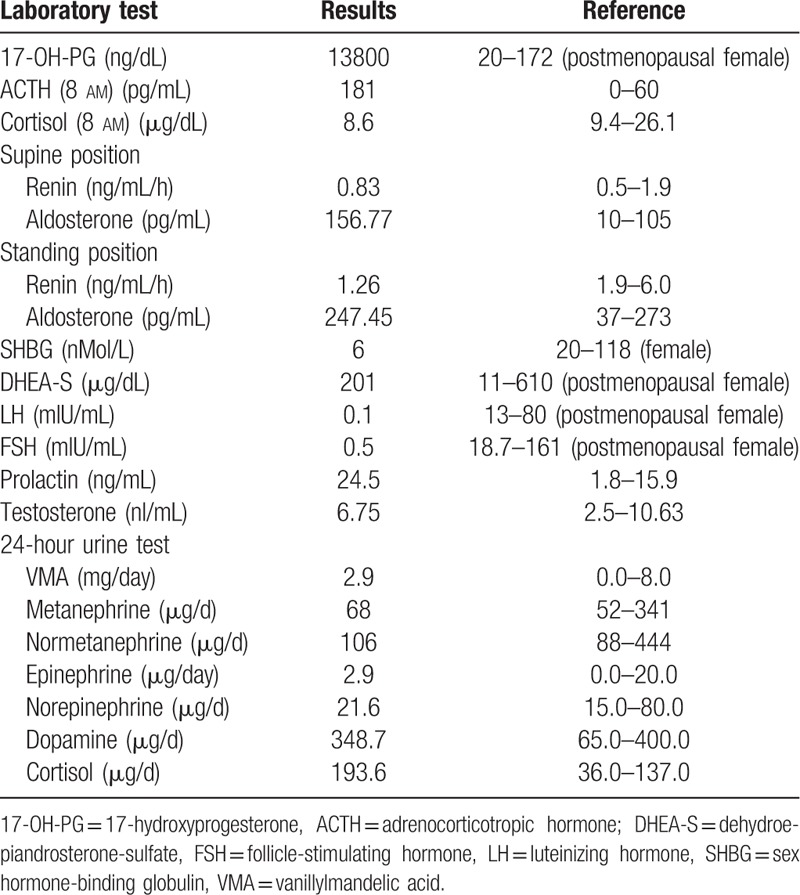
Hormone study.

**Table 2:**
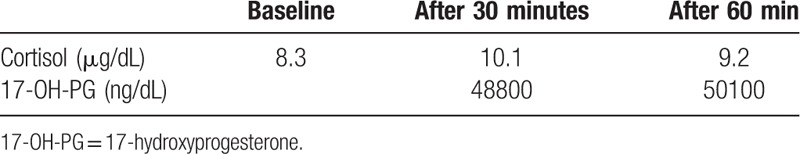
Changes in cortisol and 17-hydroxyprogesterone levels after adrenocorticotropic hormone stimulation.

On the 12th day after admission, CRP decreased to 0.12 mg/dL, and intravenous antibiotics were suspended. Positron emission tomography-CT (PET-CT) was performed to evaluate whether the mesenteric mass was malignant. PET-CT demonstrated

**Figure 2:**
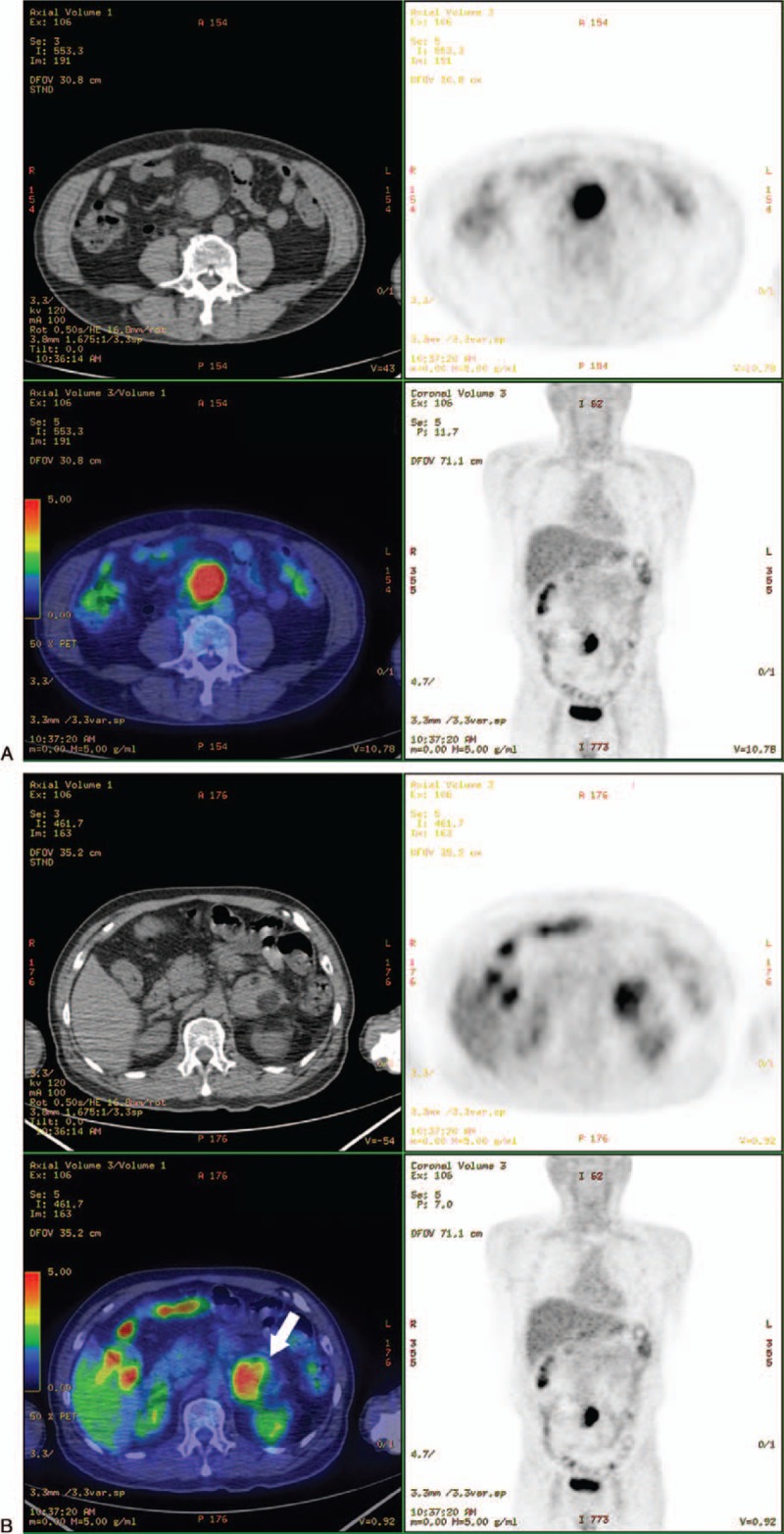
Positron emission tomography-computed tomography showing (A) A hypermetabolic mass in the mesentery suggesting primary mesenteric tumor (peak standardized uptake value [SUV] = 11.8); (B) A hypermetabolic mass in the left adrenal gland suggesting benign adrenal gland tumor (peak SUV = 5.5) (arrow).

(1) a hypermetabolic mass with homogenous, intense fluorodeoxyglucose (FDG) uptake in the mesentery suggesting primary mesenteric tumor (peak standardized uptake value [SUV] = 11.8);
(2) a hypermetabolic mass with heterogenous FDG uptake in the left adrenal gland suggesting benign adrenal gland tumor (peak SUV = 5.5) (Fig. 2).

The patient opted for resection of the female genitalia along with the mesenteric and adrenal gland tumors in the near future. The physicians planned to administer stress-dosing corticosteroid during operation, and to decrease the dose to physiologic level afterward. The patient was safely discharged. Patient has provided informed consent for publication of the case.

## 2 Discussion

CAH is divided into classic CAH and non-classic CAH. Classic CAH is further divided into the salt-wasting form and the simple virilizing form. CAH with the classic salt-wasting form usually manifests as symptoms of salt wasting and adrenal crisis within the first few weeks after birth. In the simple virilizing form of CAH, the degree of virilization varies from simple clitoromegaly to the presence of a penile urethra.^[6]^ Patients with simple virilizing CAH have 1% to 2% of 21-OH activity and, consequently, produce minimal aldosterone.^[2]^ Serum renin and aldosterone levels were normal in this patient, preventing salt wasting and also delaying the diagnosis. We suspected CAH from the patient's history, although he was phenotypically male. Moreover, the prompt decision to perform a CT scan was helpful to diagnose PID and other accompanying issues, that is, adrenal gland myelolipoma and mesenteric tumor. Of note, the 24-hour urine cortisol level was elevated despite the patient having an adrenal insufficiency. Immunoassay, which is relatively nonspecific compared to structure-based assays, was used to measure urine cortisol; however, the possibility of cross-reaction with non-cortisol substances leading to an overestimation of urine cortisol could not be excluded. ^[7,8]^ Gene mutation test for 21-OH deficiency could not be performed because of patient refusal, although it was not an obligatory test for diagnosis of CAH according to the current guideline. ^[1]^

There are several case reports that describe increased incidence of adrenal myelolipoma in CAH patients. ^[9,10]^ Further, up to 10% of myelolipoma patients have been reported to have CAH.^[11]^ The possible mechanism underlying this association is based on the exposure to a chronically excessive level of ACTH leading to the development of adrenal myelolipoma.^[3,9,10]^ This is supported by the fact that many myelolipoma patients with CAH are untreated or have poorly controlled CAH. ^[10]^ The current patient also had a very high level of ACTH and chronic dyspepsia for years. To date, there is no specific guideline regarding the management of large adrenal myelolipomas. Spontaneous rupture of myelolipomas has been reported, and massive bleeding occurred in cases with myelolipomas larger than 10 cm in size.^[11]^ The physicians and the patient in the present case discussed this issue thoroughly and decided to surgically remove the myelolipoma considering its risk of bleeding.

There are controversies regarding how to correct ambiguous genitalia after adolescence. ^[10,12]^ However, the Clinical Practice Guideline by the Endocrine Society suggests that if patients are raised as men, surgery for removal of the uterus and ovaries before puberty might be needed, considering their identity as males. ^[1]^ In the case of our patient, we decided to remove the uterus, ovaries, and vaginal stump because the surgery for small bowel mesenteric tumor was inevitable and the remaining female genitalia could cause recurrent PID.

To date, this is the first case report of a primary mesenteric tumor in a CAH patient. Further reports to investigate an association between mesenteric tumors and CAH are required.

In conclusion, male patients who were born before the era of mandatory neonatal screening for CAH, who are suspected to have CAH from their history, and present with abdominal pain need to be evaluated by imaging study and serum 17-OH-PG level examination to determine the presence of counter-sex internal genitalia and adrenal gland neoplasm.

## Acknowledgments

We would like to thank Editage for English language editing.

## Author contributions

**Conceptualization:** Eunsoo Lim, JA Young Jeon.

**Data curation:** Eunsoo Lim, JA Young Jeon.

**Formal analysis:** Eunsoo Lim.

**Investigation:** Eunsoo Lim.

**Methodology:** Ja Young Jeon.

**Supervision:** Ja Young Jeon.

**Visualization:** Eunsoo Lim, Ja Young Jeon.

**Writing – original draft:** Eunsoo Lim.

**Writing – review & editing:** Eunsoo Lim, Ja Young Jeon.

Ja Young Jeon orcid: 0000-0002-3877-0479.
